# Impact of Chewing Bags, E-Cigarettes, and Combustible Cigarettes on Arterial Stiffness and Small Airway Function in Healthy Students

**DOI:** 10.3390/toxics11010077

**Published:** 2023-01-14

**Authors:** Annabelle Susann Hauck, Isabel Buchwald, Henrik Watz, Frederik Trinkmann, Charlotte Söling, Andrea Rabenstein, Tobias Ruether, Kai Mortensen, Daniel Drömann, Klaas Frederik Franzen

**Affiliations:** 1 Medical Clinic III, Site Lübeck, University Hospital Schleswig-Holstein, 23562 Luebeck, Germany; 2 Airway Research Center North (ARCN), German Center for Lung Research (DZL), 23562 Luebeck, Germany; 3 Pulmonary Research Institute (PRI) at LungenClinic Grosshansdorf, 22927 Grosshansdorf, Germany; 4 Thoraxklinik Heidelberg, Translational Lung Research Center Heidelberg (TLRC), German Center for Lung Research (DZL), University Hospital Heidelberg, 69120 Heidelberg, Germany; 5 Department of Biomedical Informatics, Heinrich-Lanz-Center, University Medical Center Mannheim, Heidelberg University, 69120 Heidelberg, Germany; 6 Klinik für Psychiatrie und Psychotherapie, LMU Klinikum, 80336 Munich, Germany; 7 Cardiology Kiel, 24116 Kiel, Germany; 8 Clinic for Rhythmology, Campus Lübeck, University Hospital Schleswig-Holstein, 23562 Lubeck, Germany

**Keywords:** chewing bag, snus, small airway function, arterial stiffness, oscillometry, chronic lung disease, smoking cessation

## Abstract

Several substitute products are discussed as a healthier alternative to smoking, thereunder e-cigarettes and smokeless tobacco products, e.g., chewing bags, which are increasingly used in this context. We investigated the acute effects of chewing bags compared to combustible cigarettes and e-cigarettes with and without nicotine on small airways and arterial stiffness in a head-to-head design. This single-center, four-arm cross-overstudy included 20 healthy occasional smokers (25 ± 0.6 years). On four test days, participants consumed one product per day. Before, during, and after consumption, peripheral and central hemodynamic as well as arterial stiffness parameters were measured by Mobil-O-Graph™ (I.E.M., Germany). Resistance and small airway function were assessed by tremoFlo® c-100 (THORASYS Thoracic Medical Systems Inc.). The combustible cigarette and the e-cigarettes with and without nicotine significantly increased the resistance of the small airways (*p* < 0.05), while chewing bags had no effect. All nicotine containing products (e-cigarette with nicotine, combustible cigarette, chewing bag) as well as the e-cigarette without nicotine significantly increased parameters of hemodynamic and arterial stiffness. Changes in blood pressure and arterial stiffness were similar after smoking, vaping, and using chewing bags. We conclude that e-cigarettes and combustible cigarettes have similar acute harmful effects on small airway dysfunction. All nicotine containing products are associated with an increased cardiovascular risk compared with no product use.

## 1. Introduction

Currently, several substitute products are being discussed as a healthier alternative to smoking, thereunder e-cigarettes and chewing bags. In Europe and in the U.S., chewing bags are becoming more and more popular in this context [1,2]. At the moment, chewing bags and nicotine pouches are banned in Germany because of health concerns [3]. In Sweden and the Scandinavian region, the product snus and its related product chewing bags have been widely spread for decades [4,5]. Snus, as well as chewing bags, are smoking-free tobacco products that contain predominantly tobacco, salt, water, and humectants and is available in different flavors. It is applied either as loose-ground tobacco or packed in sachets and is placed on mouth mucosa, most commonly behind the upper lip [6]. Sweden shows, compared to other countries with a tobacco history, continuously very low numbers of active smokers and lung cancer [5,7]. Nevertheless, harmful ingredients such as nicotine and cancer-causing tobacco-specific nitrosamines (TSNAs) are found in the product. The TSNA dosage varies by brand and country as it is related to manufacturing and storage [4]. TSNAs are often associated with oral cavity and esophageal cancer [8]. Several studies could not provide evidence for this assumption in snus [9]. Regarding the cardiovascular risk of the product, different results are found in numerous studies. Some claim that snus increases the risk of cardiovascular death [10], whereas other studies could not confirm this thesis [11]. There is little evidence for health effects on the respiratory tract caused by snus [4]. In contrast, Gudnadóttir et al. show an increased risk of asthma and other respiratory symptoms among snus users [12].

Smoking causes many and severe, especially pulmonary and cardiovascular, health problems. In Germany, smoking-related diseases make up a huge sum of healthcare costs [13] and every day approximately 350 deaths are caused by the consequences of smoking [14]. Therefore, a harm classification of chewing bags should be of interest and if the product can be recommended for smoking cessation. We investigate the acute effects of chewing bags compared to combustible cigarettes and e-cigarettes with and without nicotine on small airway function and arterial stiffness in a head-to-head design.

## 2. Materials and Methods

### 2.1. Study Cohort and Design

In this single center, four-arm cross-over study, 10 female and 10 male occasional smokers with a mean age of 25 ± 0.6 years are included (Table 1). The participants are recruited among students at the University of Luebeck. Before the final inclusion, participants are screened for exclusion criteria: (i) non-smokers; (ii) obesity; (iii) pregnancy; (iv) mental disorders; (v) cardiovascular disease; (vi) pulmonary disease; (vii) age < 18 years; and (viii) abnormal physical examination. To follow the guidelines for measurement of arterial stiffness [15], participants are instructed neither to drink alcohol nor to smoke conventional cigarettes, e-cigarettes, or consume chewing bags 48 h prior to every measurement. Test days have to be scheduled at least 48 h apart in order to provide a washout period. After a consideration time of at least 24 h, checking all of the criteria and agreement of the participant, a consent form is signed by the participant and the examiner. The local ethics committee agreed on the study and included it on DRKS (DRKS00020446).

**Figure 1:**
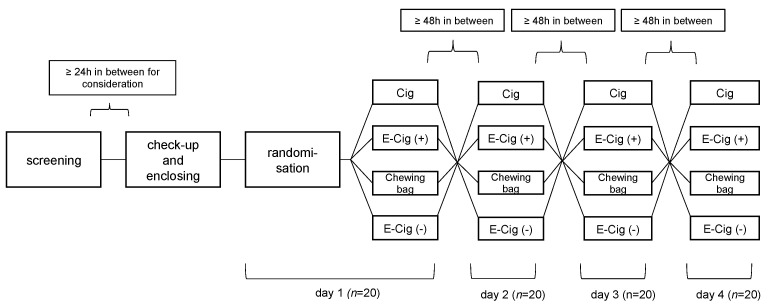
Flowchart and study design. After screening and enclosing the randomization decided the order of the four different test devices. Each device is only used once. In between the test days a washout period of at least 48 h had to be completed.

The 4 different study arms are composed of (a) cigarette (Cig) (Malboro Gold 0.5 mg/cigarette), (b) e-cigarette with nicotine (E-Cig (+)) (DIPSE-eGo-cigarette; 20 mg/mL, tobacco flavor), (c) chewing bag (Chewing bag) (Thunder Frosted Slim White Dry 8.8 mg/bag), and (d) e-cigarette without nicotine (E-Cig (-)) (DIPSE-eGo-cigarette; 0 mg/mL, tobacco flavor). Figure 1 displays the flowchart of the study.

The order of the test devices is randomized by a blinded pick of notes which each have one of the four options written on it. This way, neither the investigator nor the participant has any influence on the experimental course of action. Every test device is only used once in this study; double usage is not possible. For each of the test devices the probands are introduced to the usage. There is a clear smoking scheme for every device in order to standardize its use. Cigarettes are to be smoked until fully finished. E-cigarettes (+) and (-) are to be vaped ten times for 3–5 s with a rest period of 30 s in between. This process was chosen on the basis of previous studies [16]. Chewing bags are to be placed underneath the upper lip for 5 min. In order to complete the study and be taken into the analysis, all four conditions have to be completed as described above.

Baseline cardiovascular measurements are started 30 min prior to the intervention. The Mobil-O-Graph™ (I.E.M., Stollberg, Germany) [17,18] is programmed to a continuous measurement rhythm every 5 min. The tremoFlo® c-100 (THORASYS Thoracic Medical Systems Inc., Montreal, QC, Kanada) [19,20] is used two times prior to the intervention for baseline pulmonary measurement and four times afterwards.

In order to rule out any systematic errors in circadian rhythm, all four test days are held at the same time of the day.

### 2.2. Measurement of Peripheral and Central Blood Pressure and Arterial Stiffness

In this study, the Mobil-O-Graph^TM^ (software version HMS CS 4.2, I.E.M. GmbH), which operates with the oscillometric measuring technique, is utilized for the measurement of peripheral and central blood pressure as well as arterial stiffness parameters [17,21]. A standard blood pressure cuff is placed over the right or left arteria brachialis [18]. The ARCSolver transfer function processes the brachial waveforms, which are recorded at the level of diastolic blood pressure with the arm cuff, to the central systolic blood pressure. The recorded central waveforms facilitate the pulse waveform analysis. Thereby, the augmentation index and augmentation pressure are calculated [22]. Probands are measured in a seated position throughout the whole testing procedure. To acquire reference data for each individual, measurements are started 30 min prior to the intervention in a 5-min rhythm. For statistical analysis, the value prior to the intervention, referred to as the resting value, is used as a reference.

### 2.3. Measurement of Resistance and Reactance in Central and Small Airways

Lung function parameters are performed with an oscillometry instrument, the tremoFlo® c-100 (THORASYS Thoracic Medical Systems Inc.). Using airwave oscillometry (AO), where frequencies of oscillation reach from 5 to 37 Hz and are therefore higher than frequencies in normal tidal breathing, resistance (R) and reactance (X) are calculated. The resistance represents the ratio of the pressure decline along an airway segment divided by the flow velocity along this section. The reactance represents a complex term including elements which determine the lung compliance as well as inertive forces of the movement of the air column in the conducting airways. AX is defined as the reactance area. Resistance as determined at 5 and 20 Hz represents total and central airway resistance, respectively. The calculation of the difference is defined as R5-20 resembling peripheral airway obstruction. Tidal volume (VT) represents the amount of air between inspiration and expiration [19,20,23]. The participant is placed in a seated, upright position and is facing slightly upwards. A nose clip seals the nasal passage, hands support the cheeks on both sides, and the lips enclose the mouthpiece. The average of three consecutive 20-s measurements during tidal breathing is used for analysis. The value prior to the intervention, referred to as the resting value, is used as a reference for statistical analysis. For quality control reasons, the coefficient of variation (CV) ought to be lower than 15% [19].

### 2.4. Statistical Analysis

SPSS statistical software (SPSS 23 Inc., Chicago, IL, USA) is used for statistical analyses, and Sigma Plot 8.0 (Systat Software Inc., San Jose, CA, USA) is utilized for editing graphs. For statistical references of blood pressure, heart rate, arterial stiffness parameters, lung resistance, and reactance, baseline mean values are applied. The measurement prior to the intervention is referred to as the baseline value. All of the above-mentioned are tested for normal distribution by Kolmogorov–Smirnov tests. The crossover design is used as the basis for the decision to calculate a two-way repeated measures ANOVA to estimate an interaction between the type of device used and time. If there is an interaction found, the post hoc test (Bonferroni) is applied. Student’s *t*-test was used to evaluate differences between continuous baseline characteristics between groups. To individually analyze differences at the various time points between the four devices, ANOVA is used. Where appropriate, a multivariate analysis of variance (MANOVA) is applied to level age, mean arterial pressure (MAP), heart rate (HR), and sex. All data are expressed as mean ± standard deviation (SD) unless otherwise identified. An alpha error below 5% was considered statistically significant.

## 3. Results

### 3.1. Cig, E-Cig (+) and (-) and Chewing Bag Show Similar Effect on Blood Pressure and Heart Rate

**Figure 2:**
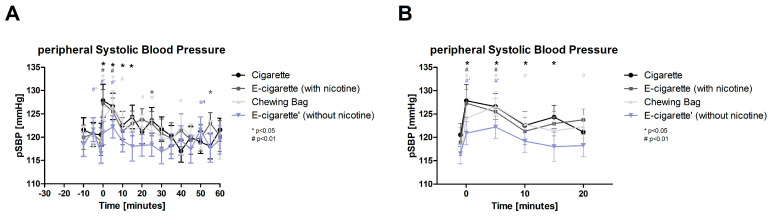
Peripheral systolic blood pressure (**A**) total follow-up; (**B**) follow-up of expected nicotine half-life. The figure displays the change in the pSBP over the time of the testing period. Four groups that are normally distributed show a significant raise at the intervention (time 0) and 5 min after. # displays a *p*-value of *p* < 0.01 (highly significant), * a *p*-value of *p* < 0.05 (significant).

All parameters are taken prior to the intervention as well as 20–40 min after the intervention at resting levels each. The peripheral systolic blood pressure (pSBP, Figure 2, normally distributed) shows an immediate significant increase after the intervention in all four devices. For the Cigarette with *p* < 0.05 and for the other three devices with *p* < 0.01. The Cig maintains the raise for 20 min, the Chewing bag for 15 min. With 7% the E-Cig (+) has the highest immediate increase; incorporating a 5-min latency, the Chewing bag shows the biggest increase with 8.5%.

**Figure 3:**
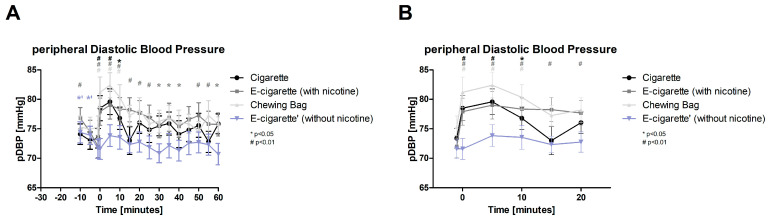
Peripheral diastolic blood pressure (**A**) total follow-up; (**B**) follow up of expected nicotine half-life. The figure displays the pDBP over time. At time 0 the intervention is undertaken, which causes the Cig, E-Cig (+) and Chewing bag to raise highly significant (# *p* < 0.01) over 15 min, Cig at 15 min after with *p* < 0.01.

After the intervention, the peripheral diastolic blood pressure (pDBP, Figure 3, normally distributed) significantly increases with a *p*-value of <0.01 for Cig, E-Cig (+), and Chewing bag instantly and stays elevated for a period of 15 min. The E-Cig (+) stays elevated the longest with 40 min. The E-Cig (+) demonstrates the biggest increase with 9.7% 5 min after the intervention, followed by the Chewing bag with 9.5%. Initially and 5 min after the intervention, the 4 groups showed a significant difference from each other (ANOVA *p* < 0.05).

**Figure 4:**
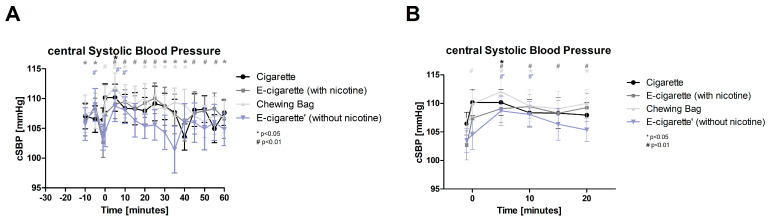
Central systolic blood pressure (**A**) total follow up; (**B**) follow up of expected nicotine half-life). Figure 4 show the cSBP over time during the testing period. At the intervention (time 0) only Chewing bag increases highly significant (# *p* < 0.01). 5 min after the intervention a highly significant (# *p* < 0.01) increase in E-Cig (+), Chewing bag and E-Cig (-) and significant (* *p* < 0.05) increase in Cig is found. The E-Cig (+) and the Chewing bag stay significantly elevated over a long period.

For the central systolic blood pressure (cSBP, Figure 4, normally distributed), a significant rise in all devices with *p*-value < 0.01 and for Cig with *p*-value < 0.05 is found 5 min after the intervention and, except for the Cig, also 10 min after. The values for E-Cig (+) stay elevated significantly for 20 min. After a latency of 25 min to the intervention E-Cig (+) shows the highest increase with 7% followed by Chewing bag with 6.5% after 5 min. With less than a 1% increase, E-Cig (-) shows a very slight change related to the intervention.

**Figure 5:**
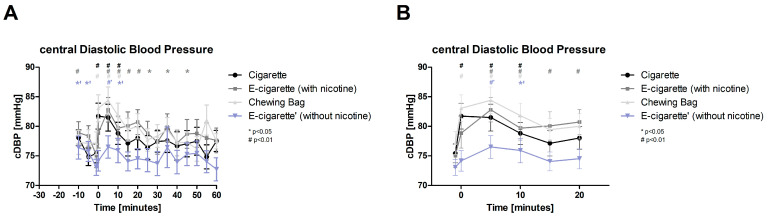
Central diastolic blood pressure (**A**) total follow-up; (**B**) follow-up of expected nicotine half-life. The cDBP plotted against time shows a highly significant (# *p* < 0.01) immediate increase (time 0) in Cig and Chewing bag. All four test groups, normally distributed, display a highly significant (# *p* < 0.01) increase at 5 min and 10 min after the intervention (E-Cig (-) significant with * *p* < 0.05). E-Cig (+) stays significantly elevated over a long period.

Initially after the intervention only Cig and Chewing bag show a significant increase (*p* < 0.01) in central diastolic blood pressure (cDBP, Figure 5, normally distributed), 5 and 10 min after the intervention all four test devices display significant values with *p* < 0.01, E-Cig (-) *p* < 0.05 at 10 min. The E-Cig (+) keeps the alteration significant over 40 min and shows the highest change 5 min post intervention with 10.3%. Followed by the Chewing bag 5 min post with 9.4%. A significant difference in between all four groups can be seen directly after the intervention (ANOVA *p* < 0.05).

**Figure 6:**
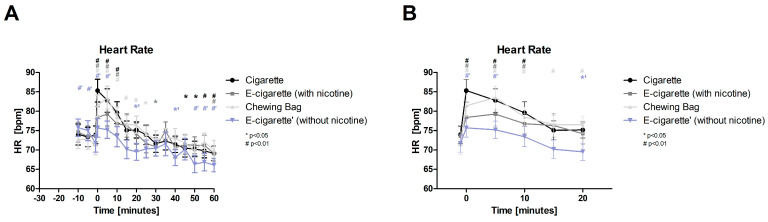
Heart Rate (**A**) total follow-up; (**B**) follow-up of expected nicotine half-life. These figures show the HR over the time of the test day. At the intervention (time 0) and 5 min after, all four groups, normally distributed, show a highly significant (# *p* < 0.01) raise; at 10 min the increase persists in all groups except for E-Cig (-).

After the intervention, the heart rate (HR, Figure 6, normal distributed) of all 4 devices raise immediately significantly with a *p* < 0.01 and last for 10 min, except for E-Cig (-) 5 min. With 15.5%, the Cig shows the highest immediate increase, followed by the Chewing bag with 14% and 17% after 5 min.

### 3.2. Cig, E-Cig (+) and (-) and Chewing Bag Show Similar Effect on Arterial Stiffness

**Figure 7:**
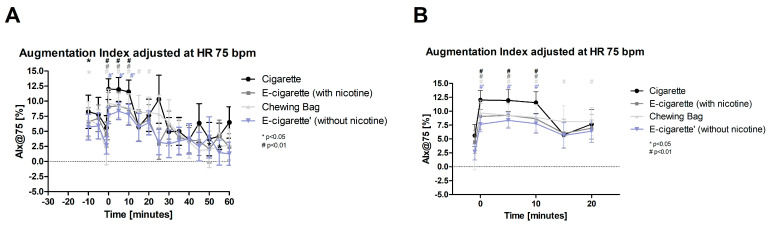
Augmentation index adjusted at HR 75 bpm (**A**) total follow-up; (**B**) follow-up of expected nicotine half-life). These figures display the Alx@75 over the time of the testing period. At the intervention (time 0), the four test groups show an immediate highly significant increase (# *p* < 0.01) which lasts until 10 min after the intervention.

The augmentation index adjusted at HR 75 bpm (AIX@75, Figure 7, normal distributed) shows a significant increase instantly after the intervention and lasts 10 min in all four test devices (*p* < 0.01). The Chewing bag stays elevated until 20 min post intervention and shows the highest increase post compared to prior-to intervention with 547%. E-Cig (-) shows an increase of 189%, the Cig 114%, and the E-Cig (+) 102%.

The Chewing bag and the E-Cig (-) both display significant changes in peripheral pulse pressure (pPP, normally distributed) after the intervention, which last throughout the whole observation period of 60 min after the intervention for the Chewing bag (*p* < 0.01 or *p* < 0.05). The Chewing bag also shows the highest increase in values with a maximum of 25% 25 min post intervention. E-Cig (-) reaches a maximum increase of 20% after 5 min, and the Cig reaches a maximum of 9% after 15 min. The value prior to the intervention shows a significant ANOVA calculation with *p* < 0.01, indicating a significant difference between the four groups.

The total peripheral resistance/vascular resistance (TVR, normally distributed) initially shows an increase for the Cig (*p* < 0.05) and the Chewing bag (*p* < 0.01). With a latency of 5 min, the E-Cig (+) and (-) and the Chewing bag demonstrate significant values with a *p*-level of <0.05. The Chewing bag shows the highest increase with 10.6%, followed by the Cig with 8.7%. Immediately after the intervention, there is a significant difference between the four groups (ANOVA, *p* < 0.05).

### 3.3. No Harmful Short-Term Effects on Small Airway Function by Chewing Bags

**Figure 8:**
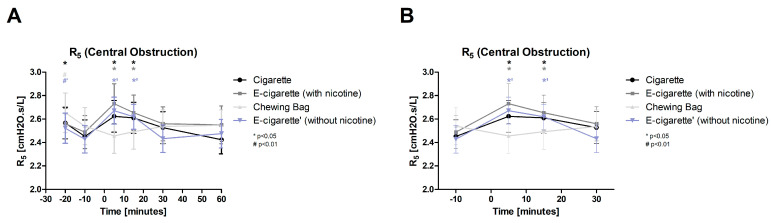
R5 (Central obstruction: (**A**) total follow-up; (**B**) follow-up of expected nicotine half-life). The central obstruction is displayed over time. The first (time 5) and second (time 15) measurements after the intervention Cig, E-Cig (+) and E-Cig (-) show a significant (* *p* < 0.05) increase. The Chewing bag stays at resting levels.

The central obstruction (R_5_, Figure 8, normal distributed) shows significant increases immediately after the intervention in all inhalation devices (Cig, E-Cig (+) and (-)) with *p* < 0.05. The significant elevation lasts until 15 min post-intervention. There is no significant change in the Chewing bag. The E-Cig (-) elevates the highest with 10%, followed by the E-Cig (+) with 9.7% and the Cig with 7%. The Chewing bag displays a decrease of 3.3%. The time instantly after the intervention shows a significant ANOVA value (*p* < 0.05) indicating a difference between the four groups.

**Figure 9:**
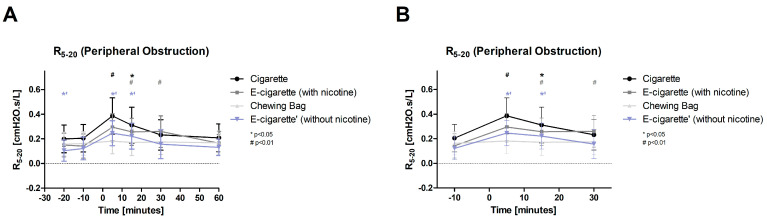
R5-20 (Peripheral obstruction: (**A**) total follow-up; (**B**) follow-up of expected nicotine half-life). In these figures, the peripheral obstruction is plotted against the time. After the intervention (time 5), Cig (highly significant # *p* < 0.01) and E-Cig (-) (significant * *p* < 0.05) showed an immediate increase. After 15 min, Cig and E-Cig (-) were significantly (* *p* < 0.05) and E-Cig (+) highly significantly (# *p* < 0.01) elevated. E-Cig (+) stays highly significantly elevated at 30 min. The Chewing bag does not show changes in values after the intervention.

Cig (*p* < 0.01) and E-Cig (-) (*p* < 0.05) significantly increase promptly after the intervention regarding peripheral obstruction (R5-20, Figure 9, normally distributed). With a latency of 15 min, all inhalation devices show a significant change (Cig and E-Cig (-) *p* < 0.05, E-Cig (+) *p* < 0.01). The Chewing bag does not show a significant change and stays at resting levels throughout the whole observation period. E-Cig (+) increases the highest with an augmentation of 107.7%, followed by the E-Cig (-) with 101.7% and the Cig with 87.9%. The Chewing bag shows an increase of 11.2%. For the measurement initially after the intervention, the four groups revealed a significant difference between each other (ANOVA *p* < 0.05).

## 4. Discussion

Even though smokeless tobacco products, especially snus, have been widely spread in Sweden for decades [4,5], they do not enjoy great popularity in most parts of Germany and the whole of Europe. Due to the low consumption rate, there are only a few studies focusing on the health effects of these products. At the current state of research, this is the first study with a German cohort, comparing a smokeless tobacco product head-to-head with three inhalation devices.

The results of our study demonstrate acute effects in blood pressure and heart rate in all four test devices, as well as for arterial stiffness parameters (AIX@75, PP, TVR). Several studies have so far found an increased risk for cardiovascular death related to snus consumption [10,13,24], others describe only non-lethal cardiovascular effects [25], and some claim to not find any correlation between snus and acute myocardial infarction [11]. As our study displays the short-term effects, it can only be hypothesized about the long-term effects. Anyway, an increased risk for cardiovascular diseases such as arterial hypertension, atherosclerosis, and myocardial infarction is well conceivable. After all, risk is supposably lower than for combustible cigarettes [4,26] and several studies could not find long term cardiovascular diseases in smokeless tobacco consumers [4,27]. Additionally, looking at Sweden, where cardiovascular health has increased within the last 25 years when people continued to stop smoking and changed to snus instead [4]. The increase in blood pressure, heart rate, and arterial stiffness parameters observed in our study can be explained by the nicotine impact. It stimulates the acetylcholine receptors and causes a reaction in the central nervous system and the vegetative nerval system. This causes the heart rate and blood pressure to rise [7]. The maximum elevation of the above-mentioned parameters can be observed immediately after the intervention, sometimes with a latency of 5–10 min. This is explained by the rapid absorption of nicotine through the lung or mouth mucosa and reaching the brain within 10–20 s [28]. As the values for the Chewing bag rise as fast as the values of the inhalation devices, it implies that the nicotine uptake through the mucosa is as fast as that through the small airways and lung alveoli. As the nicotine uptake through the buccal epithelium depends on the pH of the product, the effect could vary from brand to brand as there is no standardized pH. The more alkaline the product, the better the nicotine uptake [1]. Another observation is the slower return of Chewing bag to resting values after the intervention in several parameters (pSBP, cSBP, HR, AIX@75, pPP). This can be explained by the assumption that some of the nicotine stays in the buccal epithelium even after removing the chewing bag, thereby the effect can be monitored for a longer period [28]. At an average value return to resting levels approximately 20–40 min after the intervention. This can be explained by the half-life of nicotine and its fast distribution in tissues [28]. The Chewing bag together with the E-Cig (+) show the highest increase in values in almost all cardiovascular parameters. This can be explained by the higher dosage of nicotine in the products, as the Chewing bag contains 8.8 mg per bag and the E-Cig (+) 24 mg/mL, while the Cig only has 0.5 mg nicotine per cigarette.

Regarding the central and peripheral obstruction of the airways, our study displays that all inhalation devices, also the E-Cig (-), show a significant increase in values after the intervention. In contrast, the Chewing bag did not show any increase at all but even a reduction of obstruction in the central airways after the intervention. This could be explained by the spearmint aroma of the Chewing bag [29]. In some studies, it is stated that snus use leads to an increased risk of asthma and other lower airway alterations [12]. With the results of our investigation, we cannot prove this assumption. We rather follow Foulds et al.’s results claim that snus use leads to no or little association with pulmonary diseases and lung cancer [4,30]. Another advantage is the fact that surrounding people are not plagued by the harmful passive smoke and thereby are not put into health risk. Certainly the healthiest way is to not consume any drugs at all, but data shows that in history numbers of consuming people have been fairly steady, only with shifting in between different products [14]. Taking this information into account and considering our results, from today’s data base, chewing bags are quite certainly the healthier drug compared to combustible cigarettes and e-cigarettes with and without nicotine. Furthermore, there are new smokeless tobacco products on the market called nicotine pouches, which are available without nicotine and without tobacco. This could provide even bigger health benefits by having a possible lower effect on the vascular system [1,31], but it could also be less attractive for smoking cessation as the nicotine flash, to which many smokers are addicted, is missing. Therefore, further investigation is needed. Furthermore, most smokeless tobacco products cannot be purchased legally in Germany as there are restrictions on the distribution and difficulties from the government concerning the correct declaration of the product [7]. This way, the product will likely not reach a popularity level comparable to that of Sweden in order to convince smokers to switch to smokeless tobacco products [26].

Several considerations should be taken into account when discussing our study. One is the number of probands (*n* = 20) which is quite small. However, we used an elaborate randomized crossover design, providing sufficient power for descriptive analysis. Another is that all participants were young and healthy, and the effects could be more severe in young (<18 years), elderly, or diseased persons. Furthermore, despite standardizing the procedure of the consuming procedure, every individual has a different smoking procedure concerning the deepness of the inhalation, also due to anatomical differences, e.g., lung volumes, sex, weight. In addition, every proband consumed the same amount of the product, regardless of the different weight and body size. At this point, an adjustment could be considered for further investigations. As a further point, the cotinine concentration in the urine or blood should be collected parallel to the measurement in further studies. This would allow statistical conclusions to be drawn about the complementary effects of nicotine and, if necessary, adjust the results accordingly. Moreover, chewing bags have been used for 15–30 min in other trials. This study chose five minutes usage in order to keep it comparable with the test periods of the other test devices. There is a possibility of time dependence and influence on the investigated parameters. In addition, since this study observes individuals for only four test days within short period of time, it is not possible to achieve a databased statement on long-term and merely on acute effects. Therefore, chronic changes in cardiovascular and pulmonary parameters can only be deviated by the results but not proven. In further work, the different flavors should be compared again against each other, as there is a possibility that the different aromas could have different effects on the vessels and the small airways.

## 5. Conclusions

After comparing combustible cigarettes, e-cigarettes with and without nicotine, and chewing bags, we could show that there are harmful effects of all nicotine-containing products on blood pressure, heart rate, and arterial stiffness parameters. In contrast, lung function changes are not evident in the Chewing bag group as it is not applied via the inhaled route. Therefore, we speculate that all nicotine-containing products might have a similar cardiovascular risk profile indicative of future development of, e.g., arterial hypertension or atherosclerosis. Our finding of increased small airway resistance following the inhalation of e-cigarette vapor questions the concept of harm reduction by e-cigarettes regarding lung function abnormalities.

## Figures and Tables

**Table 1:** Specifies baseline characteristics for all subjects.

Sex	All (*n* = 20)	Male (*n* = 10)	Female (*n* = 10)	*p*-Value
Age [years]	25.0 ± 0.6	25.4 ± 3.7	24.6 ± 1.9	0.479
Weight [kg]	69.2 ± 3.0	80.5 ± 7.4	57.8 ± 5.8	<0.001
Height [cm]	177 ± 2.5	186.7 ± 5.3	167.7 ± 5.3	<0.001
BMI [kg/m²]	21.8 ± 0.5	20.5 ± 1.7	23.1 ± 2.3	0.021
Waist [cm]	74.5 ± 2.0	81.6 ± 4.3	67.4 ± 6.7	<0.001
Hip [cm]	85.2 ± 2.5	91.4 ± 9.7	79 ± 8.9	0.010
Cigarettes per day	0.77 ± 0.5	1.2 ± 3.1	0.3 ± 0.6	0.409
Fagerström Test for Nicotine Dependence [points]	0.1 ± 0.1	0.1 ± 0.3	0.1 ± 0.3	1.0

## Data Availability

Not applicable.
